# Short-Term Effects of an mHealth Intervention on Healthy Behaviors and Cardiometabolic Health in Sedentary Employees: Quasi-Experimental Study

**DOI:** 10.2196/70074

**Published:** 2026-04-27

**Authors:** Yun-Ping Lin, Shu-Hua Lu, Kwo-Chen Lee, Wei-Fen Ma, Ya-Fang Ho, Wen-Chun Liao, Hui-Ting Yang, OiSaeng Hong

**Affiliations:** 1 School of Nursing, China Medical University, 100, Sec 1, Jingmao Rd, Taichung, 406040, Taiwan, 886 422053366 ext 7125; 2 Department of Nursing, China Medical University Hospital, Taichung, Taiwan; 3 School of Food Safety, Taipei Medical University, Taipei, Taiwan; 4 School of Nursing, University of California, San Francisco, San Francisco, CA, United States

**Keywords:** sedentary work, physical activity, healthy diet, mobile health intervention, cardiometabolic health, employee

## Abstract

**Background:**

Sedentary employees face increased chronic health risks due to physical inactivity, immobility, and unhealthy eating behavior. Although mobile health (mHealth) interventions show promise in improving lifestyle behaviors, their effectiveness in occupational settings remains underexplored. Building on previous workplace interventions, this study developed and evaluated a mobile-enabled web app, SIMPLE HEALTH, developed with Din-J Design Co, Ltd, integrating activity tracking, healthy eating, and behavioral support for sedentary employees.

**Objective:**

This study evaluated the short-term effects of a 12-week mHealth intervention on physical activity, sedentary behavior, dietary habits, and cardiometabolic health indicators among sedentary employees in Taiwan.

**Methods:**

A 2-arm quasi-experimental study was conducted at 2 aerospace industrial workplaces. A total of 101 sedentary employees (mean age 46.9, SD 12.2 years; 52/101, 51.5% female) were enrolled from 2 worksites that were assigned by coin toss to either the intervention condition (n=50) or the control condition (n=51). The intervention group participated in the SIMPLE HEALTH program, an mHealth intervention grounded in Social Cognitive Theory and the Ecological Model, consisting of 8 components: activity tracking, goal setting, behavior logging, reminders, personalized advice, educational and motivational electronic booklets, and individual and team challenges. The control group received 6 print educational booklets. Cardiometabolic biomarkers, objectively measured physical activity (Fitbit Charge 3; Fitbit Inc), occupational sitting (occupational sitting and physical activity questionnaire), and dietary behavior (3-day photographic food records and the healthy eating behavior inventory) were assessed at baseline and 12 weeks. Data were analyzed using generalized estimating equations following the intention-to-treat principle.

**Results:**

At 12 weeks, the intervention group showed a significant increase in step counts (adjusted mean difference, MD 1227.13, 95% CI 2.90-2451.36; *P*=.049), a more favorable between-group change in moderate physical activity (adjusted MD 0.17, 95% CI 0.01-0.33; *P*=.04), and favorable dietary behaviors, including reduced intake of calories (adjusted MD −144.59, 95% CI −276.57 to −12.60; *P*=.03), carbohydrates (adjusted MD −19.88, 95% CI −37.99 to −1.78; *P*=.03), fats (adjusted MD −6.99, 95% CI −13.69 to −0.29; *P*=.04), and grains (adjusted MD −1.46, 95% CI −2.43 to −0.50; *P*=.003), and increased vegetable intake (adjusted MD 0.47, 95% CI 0.06-0.88; *P*=.02), compared to the control group. Favorable trends were noted in diastolic blood pressure (adjusted MD −2.38, 95% CI −4.99 to 0.22; *P*=.07) and soft lean mass (adjusted MD 0.34, 95% CI −0.06 to 0.75; *P*=.10). Both groups showed significant within-group improvements in low-density lipoprotein cholesterol (intervention: *P*=.01; control: *P*=.03), body fat percentage (intervention: *P*<.001; control: *P*=.01), waist circumference (intervention: *P*=.001; control: *P*=.002), and occupational sitting (intervention: *P*<.001; control: *P*=.03), and occupational walking (intervention: *P*=.01; control: *P*=.046), but between-group differences were nonsignificant.

**Conclusions:**

The 12-week mHealth intervention improved physical activity and dietary behaviors and showed favorable trends in cardiometabolic indicators among sedentary employees. These findings support integrating mHealth programs into employee wellness initiatives to promote healthy behaviors, mitigate productivity loss, and reduce chronic disease burden. Further research should assess long-term sustainability, scalability, and cost-effectiveness in diverse occupational settings.

## Introduction

Sedentary employees face an elevated risk of developing chronic health conditions due to physical inactivity, prolonged sitting, and poor dietary habits. In modern economies, the demand for physical labor has declined, resulting in more desk-based occupations that require employees to remain seated for extended periods. This occupational shift, compounded by the pervasive use of digital technologies, has contributed to widespread sedentary behavior in the workplace [1,2].

Furthermore, sedentary habits often extend beyond work hours, with employees spending their leisure time engaging in passive activities such as watching television, browsing social media, or using computers [3]. Globally, approximately 27.5% of adults fail to meet the World Health Organization (WHO) recommendations for physical activity [2]. In Taiwan, this issue is more pronounced—43.3% of adults do not achieve the recommended levels of physical activity [4], and 34.6% of workers report not engaging in any exercise in the past month [5]. Without effective intervention, it is projected that nearly 500 million new cases of preventable noncommunicable diseases will occur between 2020 and 2030, resulting in an annual global health care cost of approximately US $27 billion [2].

Unhealthy dietary patterns further exacerbate these risks. Office environments often promote overconsumption of calorie-dense and nutrient-poor foods, such as snacks and meals high in fat, sugar, and sodium [6,7]. In Taiwan, only 16% and 15.5% of adults meet daily vegetable and fruit intake recommendations, respectively, while average salt intake—9 g for men and 7.2 g for women—exceeds the WHO’s recommended limit of less than 5 g per day [4]. Inadequate fruit and vegetable intake is estimated to contribute to 19% of gastrointestinal cancers, 31% of ischemic heart disease cases, and 11% of strokes, with diet-related cardiovascular diseases and cancers comprising approximately 85% and 15% of the total disease burden, respectively [8]. Moreover, global analyses attribute 2.4 million deaths annually to insufficient fruit and vegetable consumption and 1.8 million deaths to excess sodium intake [9].

Considering these challenges, the adoption of healthy lifestyles is crucial in preventing chronic diseases, premature death, and mitigating social and economic burdens. A large prospective cohort study [10] in Iran with more than 50,000 participants has demonstrated that combining healthy eating with physical activity significantly reduced the risk of mortality from all causes and cardiovascular diseases. Meta-analyses have also shown that a prudent or healthy diet is linked to reduced all-cause and cardiovascular disease mortality and a lower prevalence of metabolic syndrome [11,12]. A high-quality diet could lower the risk of atherothrombotic ischemic stroke, regardless of physical activity levels [13]. Dietary fiber intake is associated with a lower risk of metabolic syndrome [14], while high carbohydrate intake is linked to a higher risk [15].

Workplace interventions promoting good nutrition and physical activity have been shown to improve work-related and health-related outcomes. Grimani et al [16] conducted a review of 14 studies on nutrition and physical activity interventions in the workplace, revealing significant improvements in absenteeism, workability, work performance, and productivity. These benefits were particularly evident when interventions were designed to target the physical work environment and organizational structure. Similarly, Glympi et al [17] explored workplace dietary interventions among office workers, categorizing them into 4 main types: web-based, food-based, information-based, and multicomponent interventions. Among these, information- and web-based interventions emerged as the most prevalent, attributed to their cost-effectiveness. In this study, interventions refer to structured, evidence-based behavioral programs designed to improve health outcomes, while approaches refer to the delivery frameworks—such as eHealth and mobile health (mHealth)—used to implement them.

The growing availability of mobile devices and smartphones has made mHealth interventions increasingly attractive for promoting healthy behaviors in workplace settings. These interventions, which may include mobile apps, web-based platforms, text messaging, wearable devices, or sensor-assisted feedback, have demonstrated effectiveness in improving physical activity, diet quality, and health outcomes in various populations [18-20]. However, relatively few have been specifically designed for or tested among sedentary workers, who face unique environmental and behavioral challenges.

Building on our previous research [21,22], we aimed to extend traditional workplace interventions to a mobile platform. Our earlier “Sit Less, Walk More” studies demonstrated that modifying the physical environment and providing printed educational materials could increase physical activity, reduce sitting time, and improve cardiometabolic health and work productivity among office employees [21,22]. Nevertheless, postintervention feedback suggested that participants sought more flexibility, personalization, and accessibility—particularly outside the workplace. These findings guided the development of an evidence-based mHealth intervention, SIMPLE HEALTH, designed to integrate physical activity, sedentary behavior reduction, and healthy eating through a mobile-enabled web app.

The SIMPLE HEALTH program builds directly upon the theoretical and practical foundation established by our prior work. It applies Social Cognitive Theory [23], Ecological Model [24], and incorporates evidence-based behavior change techniques, such as self-monitoring, goal setting, feedback, and social support. Unlike our previous environmental interventions, which relied on printed materials and workplace modifications, SIMPLE HEALTH was delivered digitally through a web app—a platform optimized for smartphones but accessible through any internet browser. The term mHealth intervention here denotes the broader behavioral program delivered via mobile technology, whereas the web app refers to the digital interface through which users accessed the program. Although a web app can technically be opened on a desktop, SIMPLE HEALTH was specifically designed for smartphone use, allowing real-time interaction, behavior tracking, and personalized reminders through LINE (LINE Corporation), a widely used instant messaging app in Asia.

While long-term behavior change is the ultimate goal of health promotion, evaluating short-term effects is a necessary first step to assess the initial efficacy, feasibility, and user engagement of newly developed digital interventions. Consistent with the CONSORT-EHEALTH (Consolidated Standards of Reporting Trials of Electronic and Mobile Health Applications and Online Telehealth) framework, short-term evaluations provide critical feedback to refine program design and optimize future long-term trials. Therefore, this study focuses on the 12-week short-term outcomes of the SIMPLE HEALTH mHealth intervention, assessing its effects on physical activity, sedentary behavior, dietary habits, and cardiometabolic health among sedentary employees. We hypothesized that, compared with a control group receiving only print educational booklets, participants in the 12-week mHealth intervention would demonstrate significant improvements in physical activity, reductions in sitting time, healthier dietary behaviors, and favorable changes in cardiometabolic biomarkers.

## Methods

### Study Design and Setting

This study used a 2-arm, parallel, quasi-experimental design to examine the short-term effects of a mHealth intervention on healthy behaviors and cardiometabolic indicators among sedentary employees. The quasi-experimental design was selected to minimize contamination between groups while preserving the integrity of intervention delivery [25]. Recruitment and baseline assessments were conducted between April and May 2020 in 2 aerospace industrial workplaces in Taiwan.

### Participants

Inclusion criteria were full-time employment in sedentary roles (sitting ≥6 hours per workday), age ≥20 years, absence of physical limitations restricting physical activity, and access to a smartphone with the LINE messaging app and an internet connection. Exclusion criteria were part-time employment, anticipated absence from work >2 weeks, relocation within 3 months, or pregnancy or planned pregnancy during the study period. A priori power analysis [26] (effect size=0.5; power=80%; correlation=0.5) indicated 48 participants per group were needed, accounting for a 20% dropout rate as suggested by prior work [21].

### Recruitment and Allocation

Approval to conduct the study was obtained from workplace senior management. Study invitations were disseminated via company websites, email, word-of-mouth, and on-site information sessions. Occupational health nurses introduced the study at both sites, and the nurse at the intervention site was trained as a research liaison using a standardized handbook.

Participants were enrolled by occupational health nurses after obtaining site-level consent from senior management. Worksite allocation to the intervention or control condition was determined by a coin toss conducted by an independent research assistant. Group assignments were concealed until completion of baseline assessments to minimize bias. Although the quasi-experimental design limited full allocation concealment, procedures were implemented postbaseline to reduce selection bias.

Participants and the site nurse were not blinded due to the nature of the mHealth intervention. However, all data collectors (except for 1 trained research assistant) and the dietitian analyzing dietary records were blinded. Statistical analyses were conducted on deidentified data to ensure analytical blinding.

A total of 101 employees were enrolled and assigned to the intervention group (n=50) or the control group (n=51). Intervention participants also received a Mi Smart Band 4 (Anhui Huami Information Technology Co) for daily activity tracking and a participant handbook.

### Procedures

Assessments were performed at baseline and 12 weeks during paid work hours. At the control site, an occupational health nurse distributed monthly educational booklets. At the intervention site, participants engaged with the SIMPLE HEALTH web app for 12 weeks. Both groups received comparable educational content, though the delivery format differed (print vs digital).

### Intervention

The mHealth intervention, SIMPLE HEALTH, was grounded in Social Cognitive Theory [23] and the Ecological Model [24], emphasizing self-regulation, self-efficacy, and multilevel behavioral influences. Program design was guided by dietary and physical activity guidelines [27-29], expert consultation, and prior participant feedback [21]. To facilitate sustainable behavior change, evidence-based behavior change techniques were systematically incorporated [30].

SIMPLE HEALTH was a smartphone-optimized web app developed through iterative testing. It included eight interactive components: (1) activity tracking, (2) goal setting, (3) behavior logging, (4) reminders, (5) personalized advice, (6) electronic educational booklets, (7) electronic motivational booklets, and (8) individual and team challenges. A summary of the intervention components is presented in Multimedia Appendix 1, with screenshots in Multimedia Appendix 2 and detailed development and usability findings reported elsewhere [31].

Intervention participants accessed educational content equivalent to that of the control group but in a digital format. They were instructed to wear the activity tracker daily, set biweekly goals, log diet and activity behaviors, and use app-based reminders. Notifications were delivered through LINE. For data security, participants created personal login credentials and linked their accounts to the study’s LINE Official Account using encrypted connections.

### Comparator

Control participants received 6 colorful print booklets over 3 months covering 2 themes—“sit less, move more” and “healthy eating.” These materials aimed to enhance knowledge, awareness, and practical skills for healthier behaviors. Control participants did not receive access to the app or activity trackers.

### Measures

To assess the short-term effects of the intervention, we measured biomarkers of glucose metabolism, lipid metabolism, blood pressure, body composition, and physical activity and dietary behavior at baseline and 12 weeks. Data collection included an on-site assessment, activity monitoring, and a self-administered questionnaire.

#### Cardiometabolic Biomarkers

Morning on-site assessments involved collecting fasting blood samples (≥8 hours) by a trained medical technologist and a nurse. Samples were analyzed at an accredited medical laboratory for glucose, insulin, triglycerides, total cholesterol, high-density lipoprotein (HDL) cholesterol, and low-density lipoprotein (LDL) cholesterol. Blood pressure was measured twice using an automated sphygmomanometer (OMRON HEM-7320; Omron Healthcare) on the right arm, following a 3‐5 minute rest. If readings differed by >10 mm Hg for systolic or >6 mm Hg for diastolic pressure, a third measurement was taken. The average of the 2 closest readings was used for analysis. Weight, BMI, waist circumference, percent body fat, and soft lean mass were measured using a body composition analyzer (model: ioi 353; JAWON MEDICAL). This device is certified by MDD/CE (93/42/EEC) CE 0197, meeting EN ISO 13485 standards for manufacture and quality. We adhered to guidelines to ensure accuracy and participant safety.

Physical activity was objectively measured using a triaxial accelerometer (Fitbit Charge 3; Fitbit Inc). Fitbit devices have been shown in systematic reviews to provide accurate step counts in free-living conditions [32,33]. Participants in both study groups were instructed to wear the Fitbit on the nondominant wrist during all waking hours, except while bathing or swimming, for 5 consecutive days (3 workdays and 2 nonworkdays).

Activity intensity was categorized into 5 levels based on established cut points derived from metabolic equivalents (METs) [34]: (1) sedentary behavior: <1.5 METs (eg, sitting, reclining, or lying down). (2) Light physical activity (lightly active): 1.5 to <3.0 METs (eg, slow walking and household chores). (3) Moderate physical activity (fairly active): 3.0 to <6.0 METs (eg, brisk walking). (4) Vigorous physical activity (very active): ≥6.0 METs (eg, running and aerobic exercise). (5) Moderate-to-vigorous physical activity: combined time spent in moderate and vigorous activities.

The Fitbit algorithm automatically recorded time in each intensity category, total steps, and daily energy expenditure. To complement device data, participants maintained a daily activity log to document their wake and sleep times, work hours, and any instances of device removal >15 minutes. They also recorded non-primary workplace work times.

Occupational sitting time was assessed by a reliable and valid instrument, the Chinese version of the Occupational Sitting and Physical Activity Questionnaire (OSPAQ) [22,35]. Participants estimated the proportion of time spent sitting, standing, walking, and performing physically demanding tasks during a typical workday in the past 7 days. They also reported the number of hours worked and days worked in the last 7 days. Time spent in each activity (min/d) was calculated by multiplying the percent time by hours worked per day.

Dietary behavior was objectively measured using food photography. Participants in both groups were asked to take pictures of their meals for 3 consecutive days (including 2 workdays and 1 nonworkday). They uploaded the images and descriptions of their meals to LINE, where a trained research assistant collected the data. A dietitian then analyzed each meal to determine calorie content, macronutrients (carbohydrates, protein, and fat), and daily food group intakes (grains, protein foods, vegetables, and fruits). Healthy eating behavior was assessed using the 12-item Healthy Eating Behavior Inventory (HEBI) [36], which was translated into Mandarin Chinese for this study. The HEBI-C demonstrated good reliability with a Cronbach α coefficient of 0.85. Each item was rated on a 5-point Likert scale (1=never, 5=very often), with higher scores indicating healthier eating behavior.

Demographic characteristics were assessed using single-item questions covering age, sex, education, marital status, working hours, and perceived health status. Perceived health status was measured with a question from the general health domain of the 36-Item Short Form Health Survey (SF-36) Questionnaire in Mandarin Chinese: “In general, would you say your health is excellent, very good, good, fair, or poor?” [37]. Additionally, participants were asked about their history of 18 common health conditions with the question, “Have you been diagnosed by a physician with any health conditions? (Response options: no, had in the past, currently have, or under medication control).” Smoking status was assessed with two questions: (1) “How would you describe your smoking habit? (Response options: never smoked, used to smoke, or currently smoke)” and (2) “If you currently smoke, how many cigarettes do you smoke on an average day?＂

### Statistical Analysis

The intention-to-treat principle was followed, where all participants were analyzed in the groups to which they were initially assigned, regardless of whether they completed the intervention or adhered to the protocol. This approach helps to minimize bias and provides a more realistic estimate of the intervention’s effect by reflecting real-world scenarios where not all participants fully comply with the study procedures [38]. Descriptive statistics summarized the characteristics of the participants. The equivalence of the 2 groups was assessed using chi-square tests for categorical variables and *t* tests for interval and continuous variables. To examine the effects of the mHealth intervention on healthy behaviors and cardiometabolic health indicators, a generalized estimating equation (GEE) model was used. GEE was chosen because it accounts for intracorrelation between repeated measurements and provides unbiased estimates despite missing data [39]. GEE models with robust estimators and unstructured correlation structures were used, adjusting for baseline potentially confounding variables. For consistency across the text and tables, the group × time interaction effect from the GEE model is reported as the adjusted mean difference (adjusted MD), representing the between-group difference in change from baseline to 12 weeks. A 2-tailed significance level was set at .05. All data analyses were conducted using SPSS version 26.0 (IBM Corp).

### Ethical Considerations

The study protocol was approved by the China Medical University and Hospital Research Ethics Center (CRREC-109‐017). Written informed consent was obtained from all participants before enrollment, detailing study objectives, procedures, potential risks and benefits, confidentiality, and voluntary participation rights. All data were anonymized using coded identifiers and stored on encrypted servers. Participants received gift vouchers valued at NT $100 (approximately US $3) after each assessment as recognition of their time. The trial was registered at ClinicalTrials.gov (NCT04347096; registered April 13, 2020).

## Results

**Figure 1.**
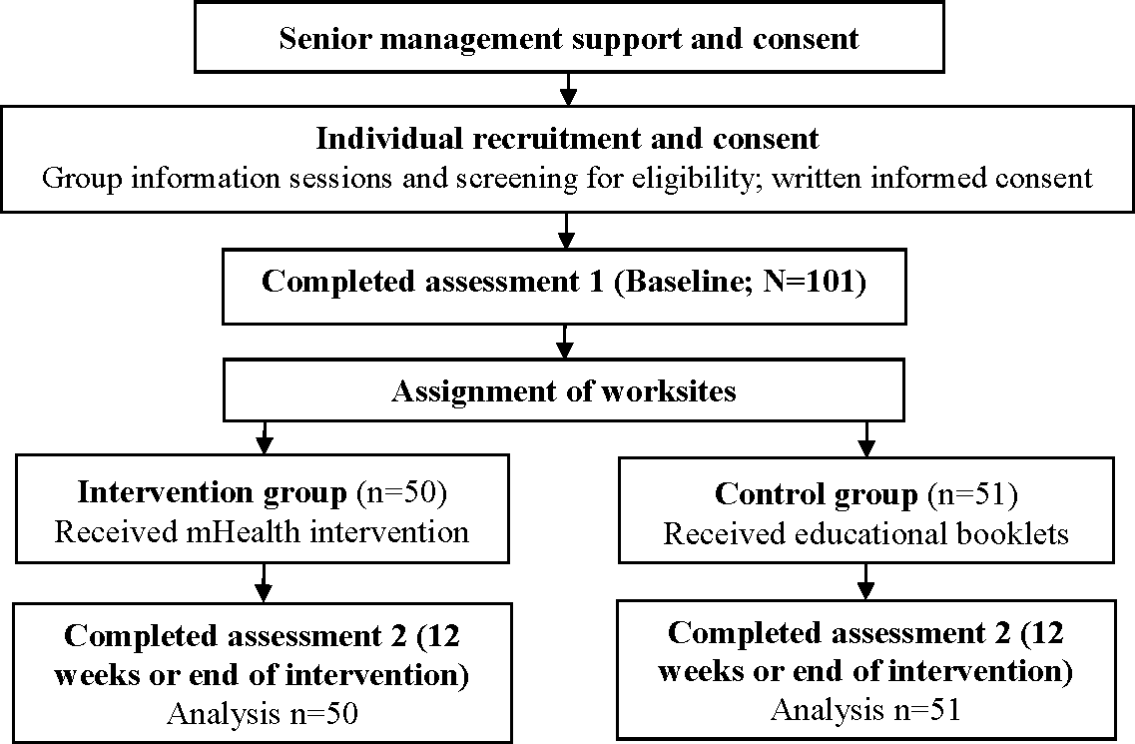
Flow diagram of participant recruitment and retention. mHealth: mobile health.

The study flow, including baseline and 12-week assessment participant numbers, is detailed in Figure 1. Demographic characteristics of the 2 groups are presented in Table 1. Both groups were similar at baseline, except for differences in age and marital status. The participants were predominantly middle-aged (mean age 46.9, SD 12.2 years), with a slight majority of females (52/101, 51.5%). Most participants were married or living together (69/101, 68.3%), had experienced common health conditions (79/101, 78.2%), had completed college or university education (47/101, 46.5%), perceived their health as fair or good (77/101, 76.2%), and were nonsmokers (88/101, 87.1%). The average weekly working hours were 46.0 (SD 2.16), and the average BMI was 24.5 (SD 4.07) kg/m².

**Table 1.** Comparison of the demographic characteristics of the participants in the intervention and control groups.

Demographic characteristics	Intervention (n=50)	Control (n=51)	*P* value^a^
Age (years), mean (SD)	50.0 (9.7)	43.9 (13.6)	.01
Sex, n (%)			.09
Female	30 (60)	22 (43)	
Married or living together, n (%)	40 (80)	29 (57)	.01
Hours worked per week, mean (SD)	45.7 (1.6)	46.4 (2.6)	.11
BMI (kg/m^2^), mean (SD)	23.8 (2.82)	25.2 (4.93)	.08
Comorbid conditions^b^, n (%)			.06
Yes	43 (86)	36 (71)	
Education, n (%)			.09
Junior college or lower	16 (32)	9 (18)	
College or university	18 (36)	29 (57)	
Graduate school and above	16 (32)	13 (26)	
Perceived health, n (%)			.35
Poor	2 (4)	4 (8)	
Fair	28 (56)	23 (45)	
Good	14 (28)	12 (24)	
Very good	6 (12)	12 (24)	
Excellent	0 (0)	0 (0)	
Smoking status, n (%)			.70
Never	44 (88)	44 (86)	
Ex-smoker	4 (8)	6 (12)	
Current smoker	2 (4)	1 (2)	

a *t* test or chi-square test between the intervention and control groups.

b Presence of at least 1 of 18 common health problems.

Table 2 summarizes the intervention effects on physical activity, sitting, and dietary behavior outcomes. The intervention group showed a significant increase in steps (adjusted MD 1227.13, 95% CI 2.90-2451.36; *P*=.049), a more favorable between-group change in moderate physical activity (adjusted MD 0.17, 95% CI 0.01-0.33; *P*=.04), and intake of calories (adjusted MD −144.59, 95% CI −276.57 to −12.60; *P*=.03), carbohydrates (adjusted MD −19.88, 95% CI −37.99 to −1.78; *P*=.03), fats (adjusted MD −6.99, 95% CI −13.69 to −0.29; *P*=.04), grains (adjusted MD −1.46, 95% CI −2.43 to −0.50; *P*=.003), and vegetables (adjusted MD 0.47, 95% CI 0.06-0.88; *P*=.02) compared to the control group (all *P<*.05). Although no treatment effects were seen for occupational sitting (intervention: baseline mean 7.90, SD 0.81 vs 12-week mean 7.32, SD 0.96; control: baseline mean 7.30, SD 1.23 vs 12-week mean 6.89, SD 1.36; mean difference −0.58, 95% CI −0.81 to −0.35; *P*<.001 vs mean difference −0.41, 95% CI −0.77 to −0.05; *P*=.03) and occupational walking (intervention: baseline mean 0.73, SD 0.66 vs 12-week mean 1.05, SD 0.76; control: baseline mean 0.86, SD 0.49 vs 12-week mean 1.07, SD 0.79; mean difference 0.32, 95% CI 0.08-0.57; *P*=.01 vs mean difference 0.21, 95% CI 0.004-0.415; *P*=.046), significant within-group improvements were observed in both groups.

**Table 2.** Effects of the intervention on physical activity, sitting, and dietary behavior outcomes.

Study outcomes	Intervention (n=50), mean (SD)	Control (n=51), mean (SD)	Group × time interaction effects in the GEE^a^ models^b^, adjusted MD^c^ (95% CI)	*P* value
Fitbit				
Steps daily				
Baseline	9975.66 (3027.42)	10530.47 (4688.18)	—^d^	—
12-week	11200.20 (3761.47)	10527.89 (4728.03)	1227.13 (2.90 to 2451.36)	.049
Lightly active (h/d)				
Baseline	4.18 (0.82)	3.95 (1.03)	—	—
12-week	4.09 (1.16)	3.93 (1.00)	–0.06 (–0.41 to 0.30)	.76
Fairly active (h/d)				
Baseline	0.39 (0.25)	0.49 (0.44)	—	—
12-week	0.36 (0.30)	0.30 (0.26)	0.17 (0.01 to 0.33)	.04
Very active (h/d)				
Baseline	0.57 (0.64)	0.56 (0.56)	—	—
12-week	0.60 (0.48)	0.46 (0.47)	0.13 (–0.11 to 0.37)	.30
MVPA^e^ (≥3.0 METs^f^; h/d)				
Baseline	0.96 (0.83)	1.05 (0.88)	—	—
12-week	0.96 (0.73)	0.76 (0.69)	0.30 (–0.07 to 0.66)	.11
Sitting time (h/d)				
Baseline	11.40 (1.46)	11.25 (1.52)	—	—
12-week	11.48 (1.72)	11.69 (1.38)	–0.40 (–0.97 to 0.17)	.17
OSPAQ^g^				
Sitting (h/d)				
Baseline	7.90 (0.81)	7.30 (1.23)	—	—
12-week	7.32 (0.96)	6.89 (1.36)	–0.17 (–0.60 to 0.26)	.44
Standing (h/d)				
Baseline	0.57 (0.64)	0.86 (0.74)	—	—
12-week	0.72 (0.44)	0.94 (0.79)	0.07 (–0.17 to 0.30)	.56
Walking (h/d)				
Baseline	0.73 (0.66)	0.86 (0.49)	—	—
12-week	1.05 (0.76)	1.07 (0.79)	0.11 (–0.21 to 0.43)	.49
Doing heavy labor tasks (h/d)				
Baseline	0.10 (0.27)	0.19 (0.41)	—	—
12-week	0.10 (0.26)	0.26 (0.70)	–0.07 (–0.27 to 0.14)	.54
HEBI^h^				
Healthy eating behavior				
Baseline	3.56 (0.44)	3.30 (0.51)	—	—
12-week	3.63 (0.42)	3.49 (0.48)	–0.12 (–0.28 to 0.04)	.14
3-day photographic food record				
Calories				
Baseline	1783.78 (245.22)	1713.15 (361.80)	—	—
12-week	1701.15 (324.95)	1775.10 (318.71)	–144.59 (–276.57 to –12.60)	.03
Macronutrients				
CHO^i^ (g/day)				
Baseline	215.70 (43.17)	204.26 (56.40)	—	—
12-week	207.91 (51.81)	216.35 (53.28)	–19.88 (–37.99 to –1.78)	.03
Protein (g/day)				
Baseline	65.93 (8.85)	63.89 (16.09)	—	—
12-week	64.58 (14.00)	62.37 (11.41)	0.16 (–5.73 to 6.06)	.96
Fats (g/day)				
Baseline	69.68 (10.63)	67.91 (15.98)	—	—
12-week	64.66 (14.17)	69.88 (14.27)	–6.99 (–13.69 to –0.29)	.04
Daily food group intakes				
Grains (portion/day)				
Baseline	10.59 (2.54)	10.25 (3.08)	—	—
12-week	10.31 (3.13)	11.43 (3.34)	–1.46 (–2.43 to –0.50)	.003
Protein foods (portion/day)				
Baseline	6.10 (1.18)	5.85 (1.96)	—	—
12-week	5.90 (1.71)	5.33 (1.44)	0.32 (–0.41 to 1.04)	.39
Vegetables (portion/day)				
Baseline	2.19 (0.97)	2.44 (1.14)	—	—
12-week	2.39 (1.19)	2.40 (1.27)	0.47 (0.06 to 0.88)	.02
Fruits (portion/day)				
Baseline	1.61 (0.95)	1.00 (1.23)	—	—
12-week	1.65 (1.14)	0.95 (1.03)	0.10 (–0.33 to 0.52)	.66

a GEE: generalized estimating equation

b Adjusted generalized estimating equations model after controlling for baseline age and sex.

c MD: mean difference.

d Not available.

e MVPA: moderate-to-vigorous physical activity.

f MET: metabolic equivalent.

g OSPAQ: Occupational Sitting and Physical Activity Questionnaire.

h HEBI: Healthy Eating Behavior Inventory.

i CHO: carbohydrate.

Table 3 summarizes the effects of the intervention on cardiometabolic biomarkers. The direction of the effects for diastolic blood pressure (adjusted MD −2.38, 95% CI −4.99 to 0.22; *P*=.07) and soft lean mass (adjusted MD 0.34, 95% CI −0.06 to 0.75; *P*=.10) favored the intervention. However, unfavorable changes were observed in high-density lipoprotein cholesterol (adjusted MD −3.99, 95% CI −5.94 to −2.06; *P*<.001) and triglyceride (adjusted MD 15.66, 95% CI 0.87-30.46; *P*=.04) levels. Within-group analysis showed that the intervention group experienced a favorable change in fasting glucose at 12 weeks (baseline mean 96.24, SD 13.66 vs mean 93.82, SD 8.70; mean difference −2.42, 95% CI −4.89 to 0.05; *P*=.06). While no treatment effects were seen for low-density lipoprotein cholesterol (intervention: baseline mean 118.76, SD 34.11 vs mean 112.97, SD 32.07; control: baseline mean 111.73, SD 24.97 vs mean 107.63, SD 22.45; mean difference −5.79, 95% CI −10.14 to −1.45; *P<*.01 vs mean difference −4.10, 95% CI −7.88 to −0.32; *P=*.03), body fat percentage (intervention: baseline mean 26.27, SD 6.03 vs mean 25.59, SD 6.00; control: baseline mean 26.02, SD 5.33 vs mean 25.55, SD 5.19; mean difference −0.68, 95% CI −1.04 to −0.33; *P<*.001 vs mean difference −0.47, 95% CI −0.85 to −0.10; *P=*.01), and waist circumference (intervention: baseline mean 80.32, SD 7.00 vs mean 79.62, SD 6.95; control: baseline mean 85.25, SD 13.77 vs mean 84.57, SD 13.51; mean difference −0.70, 95% CI −1.12 to −0.28; *P=*.001 vs mean difference −0.68, 95% CI −1.11 to −0.25; *P=*.002), significant within-group improvements were noted in both groups.

**Table 3.** Effects of the intervention on cardiometabolic biomarker outcomes.

Study outcomes	Intervention (n=50), mean (SD)	Control (n=51), mean (SD)	Group × time interaction effects in the GEE^a^ models^b^, adjusted MD^c^ (95% CI)	*P* value
Weight (kg)				
Baseline	63.56 (10.34)	69.78 (18.04)	—^d^	—
12-week	63.12 (10.42)	69.03 (17.83)	0.319 (−0.256 to 0.894)	.28
BMI (kg/m^2^)				
Baseline	23.83 (2.82)	25.25 (4.93)	—	—
12-week	23.67 (2.80)	24.98 (4.91)	0.103 (−0.120 to 0.325)	.37
WC^e^ (cm)				
Baseline	80.32 (7.00)	85.25 (13.77)	—	—
12-week	79.62 (6.95)	84.57 (13.51)	−0.024 (−0.623 to 0.576)	.94
Body fat (%)				
Baseline	26.27 (6.03)	26.02 (5.33)	—	—
12-week	25.59 (6.00)	25.55 (5.19)	−0.207 (−0.725 to 0.310)	.43
Soft lean mass (kg)				
Baseline	43.28 (8.57)	47.22 (10.79)	—	—
12-week	43.43 (8.84)	47.03 (10.71)	0.342 (−0.061 to 0.746)	.10
WHR^f^				
Baseline	0.854 (0.07)	0.863 (0.09)	—	—
12-week	0.845 (0.07)	0.858 (0.09)	−.003 (−0.010 to 0.003)	.31
SBP^g^ (mm Hg)				
Baseline	120.08 (17.56)	122.06 (14.86)	—	—
12-week	120.56 (18.98)	119.84 (13.28)	2.696 (−0.926 to 6.317)	.15
DBP^h^ (mm Hg)				
Baseline	79.54 (9.74)	82.57 (11.28)	—	—
12-week	75.98 (11.19)	81.39 (10.48)	−2.384 (−4.986 to 0.219)	.07
Glucose (mg/dL)				
Baseline	96.24 (13.66)	93.98 (12.21)	—	—
12-week	93.82 (8.70)	93.53 (16.80)	−1.969 (−5.729 to 1.791)	.31
Insulin (uIU/mL)				
Baseline	11.45 (9.03)	11.00 (7.69)	—	—
12-week	10.76 (6.90)	10.75 (6.36)	−0.428 (−2.587 to 1.731)	.70
Triglycerides (mg/dL)				
Baseline	118.88 (60.78)	117.59 (58.37)	—	—
12-week	125.72 (60.89)	108.76 (54.36)	15.664 (0.871 to 30.456)	.04
Total cholesterol (mg/dL)				
Baseline	198.96 (39.38)	187.00 (31.34)	—	—
12-week	198.56 (39.47)	186.65 (29.91)	−0.047 (−6.876 to 6.782)	.99
HDL-C^i^ (mg/dL)				
Baseline	59.66 (16.94)	51.80 (11.36)	—	—
12-week	55.74 (15.31)	51.88 (10.80)	−3.998 (−5.936 to –2.061)	<.001
LDL-C^j^ (mg/dL)				
Baseline	118.76 (34.11)	111.73 (24.97)	—	—
12-week	112.97 (32.07)	107.63 (22.45)	−1.694 (−7.451 to 4.063)	.56

a GEE: generalized estimating equation.

b Adjusted generalized estimating equations model after controlling for baseline age and sex.

c MD: mean difference.

d Not available.

e WC: waist circumference.

f WHR: waist-hip ratio.

g SBP: systolic blood pressure.

h DBP: diastolic blood pressure.

i HDL-C: high-density lipoprotein cholesterol.

j LDL-C: low-density lipoprotein cholesterol.

## Discussion

### Principal Results

This study evaluated the short-term effects of an mHealth program on physical activity, sitting time, dietary behavior, and cardiometabolic biomarker outcomes among sedentary employees. The intervention led to significant improvements in steps, moderate physical activity, and intake of calories, carbohydrates, fats, grains, and vegetables. Both the intervention and control groups demonstrated significant improvements in LDL cholesterol, body fat percentage, waist circumference, and occupational sitting and walking. However, the intervention group experienced unfavorable changes in triglyceride levels and HDL cholesterol. These findings contribute to the limited evidence on the benefits of mHealth programs targeting physical activity, sedentary behavior, and dietary habits, suggesting that such interventions may offer some cardiometabolic health benefits for sedentary employees.

### Comparison With Prior Work

The significant improvements in physical activity (steps and moderate physical activity) and dietary behavior (intake of calories, carbohydrates, fats, grains, and vegetables) observed in our study align with previous research on mHealth interventions and web-based health promotion programs targeting multiple health behaviors, including physical activity and diet [40-43]. Cook et al [40] evaluated the effectiveness of a web-based multimedia health promotion program aimed at improving dietary practices, increasing physical activity, and reducing stress in employees of a human resources company. The intervention group showed significant improvement in attitudes toward a healthy diet and dietary stage of change. Another study by Cook et al [41] examined the effectiveness of a web-based health promotion program, HealthyPast50, on diet, physical activity, stress, and tobacco use in older employees of an information technology company. The intervention group significantly improved diet behavior change self-efficacy, planning healthy eating, and mild exercise, but not physical activity. Deitz et al [42] tested the effectiveness of a web-based cardiovascular health promotion program targeting physical activity, diet, stress, mood, and smoking in hospital employees. The intervention group significantly improved dietary attitudes, intentions, self-efficacy, exercise self-efficacy, and overall level of physical activities, particularly strenuous physical activity. Finally, Oftedal et al [43] examined the preliminary efficacy of a mHealth intervention targeting shift workers’ physical activity, diet, and sleep quality. The intervention group significantly improved diet quality, though there were no significant changes in physical activity or sleep quality.

In this study, we observed inconsistent findings on the impact of increasing physical activity, reducing sitting time, and improving dietary behavior on cardiometabolic biomarkers, reflecting similar patterns found in previous research. Positive trends were noted in diastolic blood pressure, soft lean mass, and fasting glucose, though these were not statistically significant. In both intervention and control groups, there were significant improvements in LDL cholesterol, body fat percentage, and waist circumference. However, the intervention group showed unfavorable changes in triglycerides and HDL cholesterol.

The unfavorable changes in triglycerides and HDL cholesterol in the intervention group, despite promoting a low-carbohydrate diet that is high in healthy fats, may be due to several factors. Even small increases in carbohydrate intake can significantly impact lipid metabolism, potentially raising triglycerides and lowering HDL cholesterol, as the body’s lipid response is highly sensitive to carbohydrate changes [44]. The type of carbohydrates and fats consumed also matters; diets high in refined carbs or low-quality fats can negatively affect lipid profiles, even with balanced macronutrient ratios [45]. Additionally, individual metabolic responses and genetic factors can lead to varied lipid responses, further complicating outcomes [44]. In brief, these unfavorable changes likely result from subtle shifts in macronutrient intake and the types of foods consumed, underscoring the complexity of dietary impacts on lipid metabolism.

Previous research on the impact of mHealth interventions on cardiometabolic biomarkers has yielded mixed results. For example, Deitz et al [42] found no significant effects of a web-based program on waist circumference and blood pressure. In contrast, Fukuoka et al [46] evaluated a diabetes prevention intervention that combined a mobile phone app and a pedometer targeting physical activity and dietary intake in overweight adults at risk for type 2 diabetes. This intervention group showed significant reductions in hip circumference and blood pressure but no significant effects on fasting lipid levels (triglycerides, total cholesterol, LDL, and HDL) or glucose levels (hemoglobin A_1c_ and glucose).

A recent review by Jung et al [47] provided a comprehensive analysis of the effectiveness of mHealth interventions in promoting physical activity and weight loss among workers. While the mHealth intervention group significantly improved physical activity, they did not show a significant difference in weight loss. Another review by Buckingham et al [48] assessed the effectiveness of mHealth technology, including wearable activity monitors and smartphone apps, for promoting physical activity and reducing sedentary behavior in workplace settings. The review found that 69% of studies reported significant impacts on reductions in weight, systolic blood pressure, and total cholesterol.

These mixed findings highlight the complexity of mHealth interventions and their varied effects on cardiometabolic health indicators. While some studies demonstrate significant benefits in specific biomarkers, others show limited or no impact. This underscores the need for further research to identify the most effective components and contexts for mHealth interventions. This includes examining the specific features that contribute to behavioral changes and improvements in health indicators. Understanding how these interventions influence behavior and health at a physiological level can help in designing more targeted and effective programs. Furthermore, it is crucial to conduct longer-term studies to assess the sustainability of behavior changes and the long-term impact on cardiometabolic health.

Of particular interest was the finding that both the intervention and control groups showed significant improvements in LDL cholesterol, body fat percentage, waist circumference, and occupational sitting and walking from baseline to 12 weeks after the intervention. This suggests either (1) both interventions effectively improved healthy behaviors and cardiometabolic health indicators or (2) most participants in both groups were highly motivated to improve their health, contributing to the observed changes. Initially, we hypothesized that the mHealth intervention group would be more effective than the control group with print educational booklets. Since this study did not have a no-treatment control group, a cautious interpretation of the intervention effect is needed. The improvements could be attributed to assessment effects for both groups. For example, after the baseline assessment, both groups received feedback on biomarkers related to glucose metabolism, lipid metabolism, blood pressure, and body composition, as well as dietary assessment along with recommendations for calorie intake and portion sizes for the 4 food groups (whole grains, protein, vegetables, and fruits). This feedback likely served as a motivating factor and benefited the control group in addressing their health and behavioral issues. Thus, this study’s lack of a no-treatment control group limits our ability to definitively attribute the improvements to the interventions rather than other motivating factors or external influences. Future research should consider including a no-treatment control group to isolate the interventions’ effects better.

Although the control group receiving colorful print educational booklets was originally intended as a control, the scope and quality of these materials likely played a significant role in promoting behavioral regulation and self-efficacy. As a result, the print materials effectively functioned as interventions, posing a substantial rival to the mHealth program. The effectiveness of print materials in changing health behaviors and outcomes is well-supported by research [40].

Compared to the print materials, participants in the mHealth program received electronic versions of the 6 educational booklets and additional intervention components. However, the print materials offered distinct advantages: users had all the material in hand from the start without needing to access a web app and could read the booklets easily. This is particularly advantageous for middle-aged people with presbyopia, who may prefer print materials over smartphones due to the small screen size. The apparent efficacy of the print materials suggests that providing them to the mHealth intervention group could further enhance the program’s effectiveness. This finding highlights the importance of considering the format and accessibility of educational materials in health interventions, especially for populations that may have preferences or limitations related to technology use.

### Implications for Occupational Health Practice

Integrating mHealth interventions into worksite health promotion programs can benefit employee well-being and productivity. The findings from this study suggest several practical implications for occupational health practitioners:

1. mHealth technologies can potentially enhance employee engagement in health promotion activities. The convenience and accessibility of web- and smartphone-based interventions can encourage more consistent participation than traditional methods. Additionally, mHealth technologies provide real-time monitoring and feedback, helping employees set their personal goals to achieve, track their progress, and stay motivated. Personalized feedback mechanisms are essential to maximize their impact.
2. Creating a supportive environment that promotes healthy behaviors is crucial for enhancing the effectiveness of health interventions. This includes providing access to mHealth tools and resources and fostering a workplace culture that values and encourages physical activity, healthy eating, and regular health monitoring.
3. Developing educational programs that leverage both digital and print materials can cater to different employee preferences. Our study showed that print materials were effective and could complement digital interventions, particularly for employees who prefer nondigital formats. Offering a variety of educational materials ensures broader engagement.
4. Adopting a holistic approach to occupational health is essential to address multiple health behaviors simultaneously. mHealth interventions can target physical activity, sedentary behavior, diet, and other relevant health behaviors, offering a comprehensive solution for improving overall health. This approach ensures that various aspects of health are addressed together, leading to better outcomes.
5. Advocating for the integration of mHealth interventions into workplace health policies and practices is essential. Employers should be encouraged to invest in these technologies as part of their health promotion and disease prevention strategies. Integrating mHealth into the workplace can potentially sustain health improvements and cost savings in the long term.

### Strengths and Limitations

This study has several notable strengths. First, it is the first mHealth intervention study to target physical activity, sedentary behavior, and dietary behaviors in a workplace context. This comprehensive approach provides valuable insights into how these interventions can be integrated into employee wellness programs. Second, the study achieved good participant retention, enhancing the reliability of the findings. Third, using objective measures to assess most outcomes, including cardiometabolic biomarkers, physical activity, and dietary behavior, adds robustness to the results.

However, the study also has limitations. First, the relatively short duration of the intervention may not capture the long-term effects on cardiometabolic health. To mitigate this, we recommend future studies with extended follow-up periods to understand the observed improvements’ sustainability better. Second, the self-reported nature of the healthy eating behavior index and occupational sitting and physical activity questionnaire may introduce recall or social desirability bias. As we also included objective measures, the concern is minimal. Third, participant allocation was determined using a coin toss, which, although simple and transparent, is not an entirely unbiased method. Prior research has demonstrated that coin toss outcomes can be subtly manipulated or deviate from a true 50:50 probability due to human control or physical factors of the coin [49]. This limitation may have introduced a small but possible allocation bias, and future studies should use computer-generated randomization to minimize this risk. Finally, the generalizability of the results is limited as the study involved only 2 workplaces, and participants were not randomly selected. To enhance external validity, more studies with larger samples from diverse occupational settings and randomized sampling methods are recommended.

### Conclusions

The mHealth intervention effectively improved physical activity, dietary behaviors, and certain cardiometabolic health indicators among sedentary employees. These findings suggest that integrating mHealth tools into workplace wellness programs can be beneficial. Future studies should explore long-term effects and broader applications across diverse occupational settings to confirm and extend these results.

## Supplementary material

10.2196/70074 Multimedia Appendix 1 Mobile health intervention components and the behavior change techniques applied.

10.2196/70074 Multimedia Appendix 2 Screenshot of the homepage.

10.2196/70074 Checklist 1 CONSORT-eHEALTH checklist (V 1.6.1).

## Abbreviations

CONSORT-EHEALTH: Consolidated Standards of Reporting Trials of Electronic and Mobile Health Applications and Online Telehealth
GEE: generalized estimating equation
HDL: high-density lipoprotein
HEBI: Healthy Eating Behavior Inventory
LDL: low-density lipoprotein
MET: metabolic equivalent
mHealth: mobile health
OSPAQ: Occupational Sitting and Physical Activity Questionnaire
SF-36: 36-Item Short Form Health Survey
WHO: World Health Organization
