# Correction: Short-Term Effects of an mHealth Intervention on Healthy Behaviors and Cardiometabolic Health in Sedentary Employees: Quasi-Experimental Study

**DOI:** 10.2196/106593

**Published:** 2026-08-14

**Authors:** Yun-Ping Lin, Shu-Hua Lu, Kwo-Chen Lee, Wei-Fen Ma, Ya-Fang Ho, Wen-Chun Liao, Hui-Ting Yang, OiSaeng Hong

**Affiliations:** 1 School of Nursing, China Medical University, 100, Sec. 1, Jingmao Rd., Taichung, 406040, Taiwan, 886 4-22053366 ext 7125; 2 Department of Nursing, China Medical University Hospital, Taichung, Taiwan; 3 School of Food Safety, Taipei Medical University, Taipei, Taiwan; 4 School of Nursing, University of California, San Francisco, San Francisco, CA, United States

 In “Short-Term Effects of an mHealth Intervention on Healthy Behaviors and Cardiometabolic Health in Sedentary Employees: Quasi-Experimental Study” [1], the authors noted one error in Figure 1.

**Figure 1.**
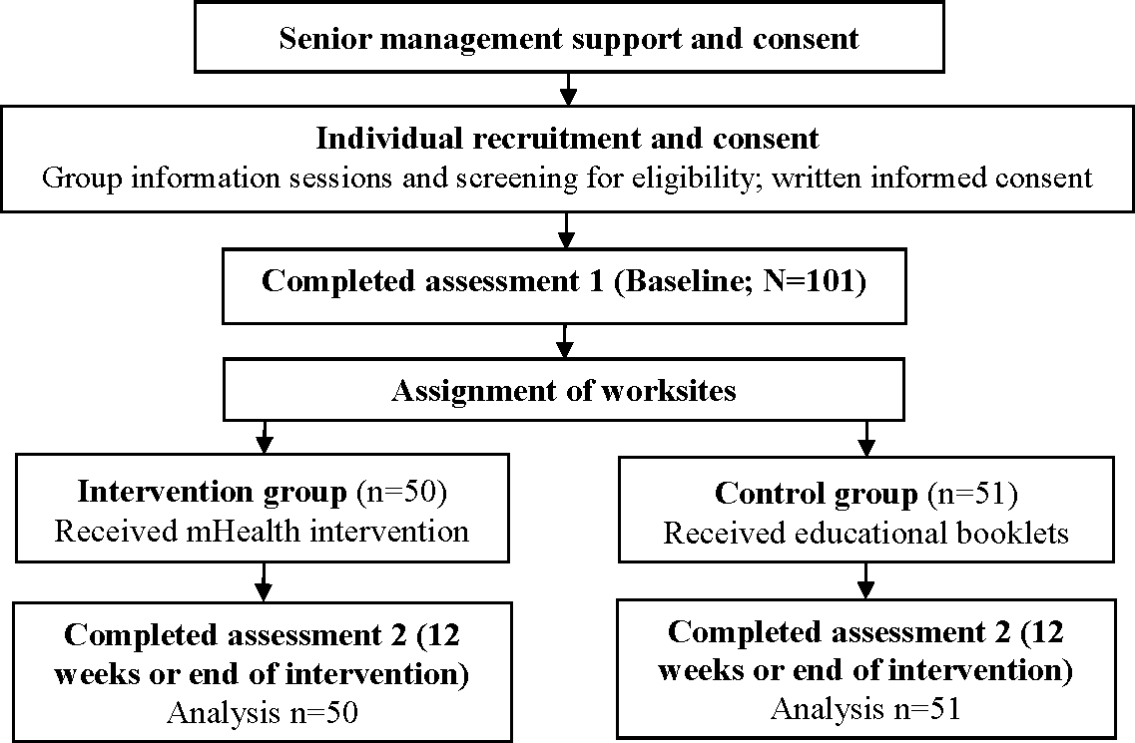
Flow diagram of participant recruitment and retention. mHealth: mobile health.

In the originally published Figure 1, the box “Assignment of worksites” appeared before “Completed assessment 1.”

The figure has been corrected so that “Completed assessment 1 (Baseline; N=101)” appears before “Assignment of worksites.” This corrected sequence is consistent with the Methods section, which states that group assignments were concealed until completion of baseline assessments.

This revision does not affect the sample size, intervention or control conditions, outcome data, statistical analyses, results, interpretation, or conclusions of the article.

The correction will appear in the online version of the paper on the JMIR Publications website, together with the publication of this correction notice. Because this was made after submission to PubMed, PubMed Central, and other full-text repositories, the corrected article has also been resubmitted to those repositories.
