# Supplementary material for: Short-Term Effects of an mHealth Intervention on Healthy Behaviors and Cardiometabolic Health in Sedentary Employees: Quasi-Experimental Study
Source: JMIR Mhealth Uhealth. 2026 Apr 27;14:e70074. doi: 10.2196/70074 (PMC13120693; doi:10.2196/70074)
Supplement: Multimedia Appendix 1 [file mhealth-v14-e70074-s001.docx]

**Multimedia Appendix 1.**  mHealth intervention components and the behavior change techniques applied.

| Component | Purpose and BCTs^a^ | Dose | What was done |
| --- | --- | --- | --- |
| Activity tracking | Activity tracking provides real-time data on steps, calories burned, and distance, helping participants monitor their physical activity goals, assess progress, and identify areas for improvement. It also offers structure, motivation, and feedback to support maintaining healthy behaviors.  BCTs: Self-monitoring, prompts/cues, habit formation. | Weeks 1–12, daily | Participants in the intervention group were given Mi Smart Band 4 activity trackers to accurately monitor their daily step count. |
| Goal setting | Goal setting provides direction and motivation, empowering participants to manage behaviors and make consistent progress. Achieving short-term goals boosts self-efficacy, encouraging continued engagement in healthy behaviors.  BCTs: Behavioral goal setting, self-monitoring, habit formation. | Weeks 1–12, biweekly | Participants set biweekly health goals through the web app, receiving guidance on creating realistic yet challenging objectives. Goals included daily calorie intake, appropriate food portions, daily step counts, and stand-up goals to move every hour. |
| Behavior logging | Behavior logging supports behavioral regulation by helping participants monitor and adjust their habits, bridging the gap between intention and action.  BCTs**:** Self-monitoring, habit formation. | Weeks 1–12, daily | Participants logged daily health behaviors—dietary intake, physical activity, and stand-up occurrences—via the web app, which also provided guidance on portion sizes for better dietary logging. |
| Reminders | Reminders act as cues to prompt specific behaviors, supporting adherence to routines and behavioral goals.  BCTs: Prompts/cues, habit formation. | Weeks 1–12, daily | Participants used the web app to set reminders for standing up and logging behaviors, with notifications sent through LINE. |
| Personalized advice | Personalized advice offers tailored guidance based on individual circumstances, making it more relevant and actionable, which supports behavior regulation and goal achievement.  BCTs: Feedback on behavior, behavior substitution, discrepancy between current behavior and goal. | Weeks 1–12, daily | Advice was customized based on each participant’s behavior and goals. Following behavior logging, participants received encouragement and reinforcement, with specific advice tailored to their progress, such as prompting more movement if sitting behavior compliance was low or reinforcing positive behavior if goals were met. |
| Electronic educational booklets | Educational booklets increase knowledge, develop practical skills, and raise awareness, motivating participants to initiate and sustain behavior changes. They promote behavioral regulation and self-efficacy by providing the knowledge and skills to manage health behaviors effectively.  BCTs: Instruction, problem solving, health consequences, behavior demonstration, credible source, action planning, behavior substitution. | Weeks 1–12, two booklets were provided monthly | Six educational booklets were developed, covering two main themes: “sit less, move more” and “healthy eating.” Topics included benefits of physical activity, tips for staying active, fundamentals of healthy eating, practical nutrition advice, safety considerations, and simple healthy recipes. |
| Electronic motivational booklets | Motivational booklets boost motivation and engagement, helping participants persist in achieving their goals by making the process rewarding and reinforcing the importance of their objectives.  BCTs: Goal setting, behavior instruction, problem solving, behavior demonstration, credible source, action planning, behavior substitution, self-monitoring. | Weeks 1–12, biweekly | Six motivational booklets were created to help participants stay motivated, overcome challenges, avoid temptations, prevent relapse, maintain enthusiasm, and sustain healthy habits, focusing on setting and achieving short- and long-term health goals. |
| Individual and team challenges | Challenges boost engagement and motivation through competition and cooperation, encouraging goal-setting and providing social support.  BCTs: Social support, social comparison, reward. | Weeks 1–12, biweekly | Participants monitored their progress and rankings via the “My Performance” section in the web app, comparing their health behaviors to peers and teams. Rankings were based on dietary habits, physical activity, and standing frequency. The top performers and team received prizes from senior management, fostering motivation and participation. |

^a^BCTs: behavior change techniques.
