# Supplementary figures and images for: Short-Term Effects of an mHealth Intervention on Healthy Behaviors and Cardiometabolic Health in Sedentary Employees: Quasi-Experimental Study
Source: JMIR Mhealth Uhealth. 2026 Apr 27;14:e70074. doi: 10.2196/70074 (PMC13120693; doi:10.2196/70074)

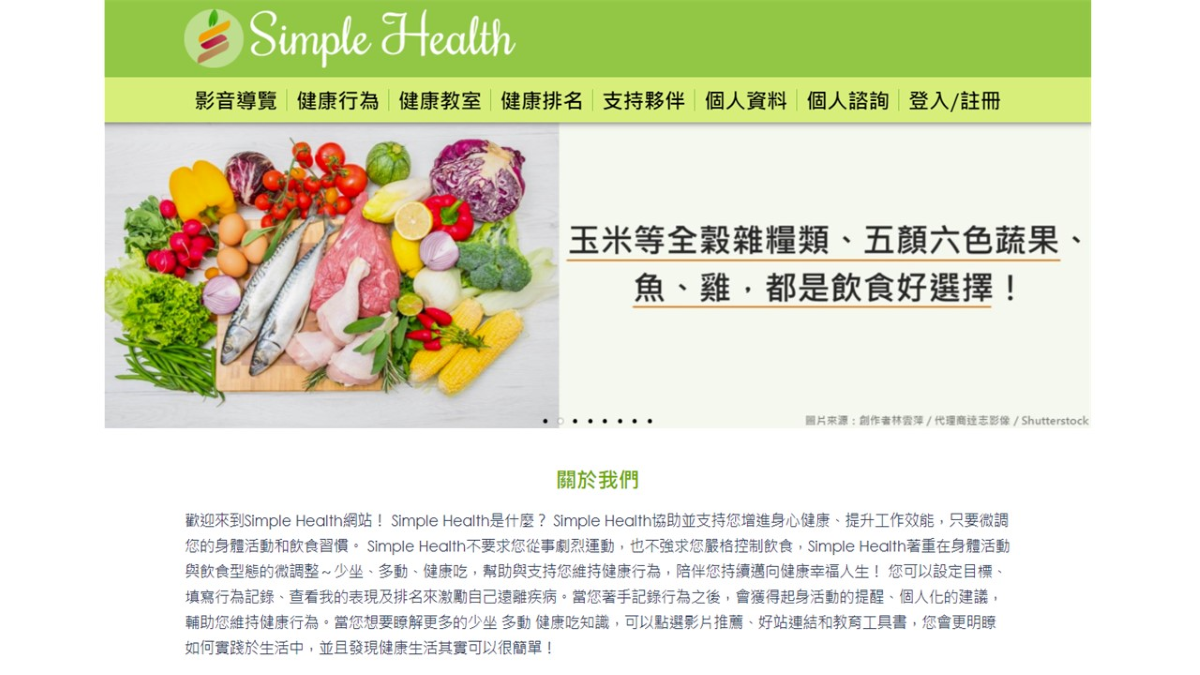

Supplement: Multimedia Appendix 2 [file mhealth-v14-e70074-s002.png]
